# Resiliency outcomes after participation in an asynchronous web-based platform for adults with neurofibromatosis: The NF-Web study

**DOI:** 10.1371/journal.pone.0295546

**Published:** 2023-12-21

**Authors:** Katherine E. Wang, Ana-Maria Vranceanu, Ethan G. Lester

**Affiliations:** 1 Center for Health Outcomes and Interdisciplinary Research, Massachusetts General Hospital, Boston, Massachusetts, United States of America; 2 Harvard Medical School, Boston, Massachusetts, United States of America; Sathyabama Institute of Science and Technology, INDIA

## Abstract

The purpose of this study was to analyze secondary resiliency and user experience outcomes from a novel, 8-week website-based mind-body intervention (NF-Web) for adults (18+) with neurofibromatosis (NF1, NF2, and schwannomatosis), a genetic, neurocutaneous disorder characterized by nerve sheath tumors of the central and peripheral nervous system. The study design was a secondary data analysis of a single-arm, early feasibility pilot study (September 2020-May 2021) for adults with NF (*N* = 28). Across participants, the mean age was 46 (*SD* = 13.67) and included 22 females and 6 males. Participants completed baseline and posttest assessments (t-tests), as well as exit interviews (exploratory rapid data analysis). Results demonstrated that participation was associated with pre-to-post improvements in gratitude, coping, and mindfulness (p < .05). Exit interviews indicated participant enjoyment and that many would recommend NF-Web to a friend. Participants found the website easy to navigate and enjoyed NF-Web’s video format. Many found transcripts useful if they had hearing differences or if English was their second language. NF-Web demonstrated initial signals of improvement in resiliency outcomes and positive user experience. Future pilot RCTs will explore these changes by NF type.

## Introduction

Neurofibromatosis (NF; NF1, NF2) is a genetic, neurocutaneous disorder characterized by nerve sheath tumors of the central and peripheral nervous system, including the brain, spinal cord, and skin [1]. NF1 is the most common, with an incidence of 1 in 3,000 births [2], followed by NF2 (1 in 33,000) [3], and schwannomatosis (1 in 60,000) [4]. Disfiguring cutaneous tumors, focal neurological deficits (e.g., poor gait, facial weakness, hearing loss), and chronic pain are common symptoms of NF1, NF2, and schwannomatosis, respectively. With no curative interventions, treatment is mostly limited to surgical and palliative options and, only more recently, pharmacological (MEK inhibitors) [5].

In light of these challenges, adults with NF can experience lower quality of life (QoL), more pain, lower self-esteem, higher levels of stress, and greater symptoms of depression and anxiety relative to the general population [6–10]. Though few NF-specific psychosocial treatment options exist, a recently developed live video intervention for adults with NF (Relaxation Response Resiliency Program for NF [3RP-NF]) was found to be feasible, acceptable, and associated with improvements in QoL, emotional distress, and pain [11]. However, exit interviews from this program indicated a need for other accessible formats of the program leveraging technology [12]. We aimed to address this identified need by developing an asynchronous website-based (“web-based”) intervention based on the feasible live video 3RP-NF program, allowing participants to complete modules on their own based on their own schedules. In general, web-based programs are useful for individuals who have barriers (i.e., transportation, mobility, schedule conflicts, learning differences, NF-specific physical appearance concerns) to traditional and live-video interventions. While web-based platforms may be a deterrent for those who do not prefer this medium for learning, expanding intervention modalities provides an opportunity to increase care accessibility. We piloted this web-based intervention in adults with NF and found it to be initially feasible and accepted by patients with positive signals of improvement for QoL and emotional distress [13].

Though NF-Web was found to be feasible, acceptable, and improved measures of QoL and emotional distress [13], resiliency outcomes (i.e., resources that make one more adaptable in the face of adversity) measured as part of the program (e.g., coping, mindfulness, optimism, gratitude) have yet to be explored. Improvements in coping, social support, and mindfulness were associated with previous live video 3RP-NF trial participation [14]. Additionally, we have yet to assess user experience and participant impressions of NF-Web which may provide valuable feedback for future web-based psychosocial programming.

We now report on our secondary analysis of resiliency outcomes, as well as qualitative findings from exit interviews, to better understand the NF-Web program outcomes and user experience. We hypothesized that participation in NF-web would be associated with improvements in resiliency outcomes (perceived social support, gratitude, optimism, perceived coping abilities, mindfulness, and empathy) pre- to post-test and that NF-web would elicit positive user impressions at time of program completion.

## Methods

### Procedures

The Massachusetts General Hospital Institutional Review Board (IRB) approved all procedures related to this study (approval number: 2019P002950). We obtained informed written consent from all individual participants prior to enrollment. Before starting the program, participants completed baseline measures through REDCap (a secure electronic research data collection software) [15] and an orientation/introduction module to guide platform usage. Following this introduction module, participants began completing weekly NF-Web modules for 8 weeks as they were assigned by study staff (i.e., unlocked on Wordpress.org).

While this study was conducted during the COVID-19 pandemic, participant experience of the platform–barring broad impacts like any other study–was largely unaffected, due to the web-based format of the program. Procedurally, from the study administrator standpoint, there were some additional challenges in recruitment, coordinating meetings, and follow up assessment; however, they were similarly mitigated by the web-based design. Throughout the program, study staff monitored module completion via the dashboard function *Users* in Wordpress.org. In order to effectively do so, we maintained access to information that could identify participants during data collection. Study coordinators contacted participants who did not complete the first NF-Web module within 4 days of the first week or missed two or more sessions consecutively to resolve any relevant technical issues or barriers. We recorded weekly module completion, discussion board participation, survey completion, and homework completion in a separate study log on a secure desktop.

After the program concluded, participants completed posttest assessments through the same REDCap system [15]. We gave participants the option to complete a semi-structured exit interview by telephone to assess satisfaction, barriers and facilitators, and suggestions for NF-Web improvements (user experience) after program completion.

### Participants

We recruited participants between 10/1/2020–5/13/2021 from two primary sources: from our efficacy trial study log of those who expressed interest but could not participate [11], and through our study flyer sent to our funding foundations (NF Northeast, NF Midwest, Texas Neurofibromatosis Foundation, and the Children’s Tumor Foundation) through emailed newsletters, foundation announcements, and websites. Beyond this recruitment contact, the funders had no role in study design, analysis, decision to publish, or preparation of this manuscript.

We successfully recruited and enrolled 28 participants across two cohorts (14 each) to take part in the 8-week NF-Web trial. Our recruitment strategy attempted to stratify by NF type to reflect the relative prevalence of each illness in the general population. Our enrollment goal was 25–30, leaving approximately 16–20 participant slots for NF1, 4–6 for NF2, and 2–4 for SCHW. When we met specific enrollment goals for an NF type, we shifted focus to enrollment of other NF types accordingly. We also used a cohort-model of participation to simulate group interactions with discussion board content and to optimize administrative support for NF-Web (e.g., website support, email contact). Inclusion/exclusion criteria are the same as the live video trial [11] and are presented in Table 1 below.

**Table 1:** Inclusion/Exclusion criteria for NF-Web.

Inclusion/exclusion Criteria for NF-Web
• Adults aged 18 years or older • Diagnosis of neurofibromatosis (NF; NF1, NF2, or schwannomatosis) • English-speaking and self-reported literacy at 6^th^ grade reading level or above • Self-reported difficulties coping with stress and NF symptoms • Score of 6 or higher on the Perceived Stress Scale v.4 (PSS-4)
• No Cognitive Behavioral Therapy or relaxation therapy in the past 3 months • No change in antidepressant medication in the past 3 months
• No medical comorbidity expected to worsen in the next 12 months
• Did not participate in previous adult live video program (3RP-NF) • Able to complete questionnaires online and participate in live video interventions

### NF-Web program

The NF-Web program is an 8-module, asynchronous web-based mind-body intervention with content adapted directly from the 3RP-NF mind-body group intervention for adults with NF [11] and designed based on qualitative information gathered from 3RP-NF participants [12]. Each week, participants complete treatment modules at their own pace over the week period. Participants are taught resiliency skills from the 3RP-NF in each session through pre-recorded video lessons by the PI (EGL), including skills such as stress awareness (e.g., identifying negative automatic thoughts) and coping strategies (e.g., adaptive thinking). Each module includes audio, video, transcripts, recordings of transcripts, summary sheets, and embedded links to other parts of the website [13,16]. Participants are also administered weekly homework assignments and quizzes through the NF-Web platform to ensure adherence and comprehension of materials. Each module includes 30 minutes of video in each session, broken down into 5–7 videos that are 3–5 minutes in length. Participant completion time per module is approximately one hour or less.

In addition to the program, participants have access to a discussion board integrated into the NF-Web webpage. Each module has a specific prompt provided by study staff to encourage participants to go over to the discussion board and connect with other participants. Participants are also provided a “Frequently Asked Questions” (FAQs) page, as well as a contact page where participants were able to ask questions directly to the study staff.

### Measures

#### Social support

The Medical Outcome Study Social Support Survey (MOS) [17] is a 19-item measure used to assess perceptions of social support within four domains: emotional, tangible, affectionate, and positive social interactions. Items on the MOS are rated on a Likert-type scale ranging from 1 (*none of the time*) to 5 (*all of the time*) with higher scores indicating greater perceived social support (range: 19–95). The alpha for this measure was .96.

#### Gratitude

The Gratitude Questionnaire (GQ-6) [18] is a 6-item measure that assesses an individual’s dispositional gratitude. Items on the GQ-6 are rated on a Likert-type scale ranging from 1 (*strongly disagree*) to 7 (*strongly agree*) with higher total scores representing a greater disposition towards gratitude (range: 7–42). The alpha for this measure was .93.

#### Optimism

The Life Orientation Test–Revised (LOT-R) [19,20] is a 10-item measure that assesses personal tendencies towards optimism vs. pessimism (bidimensional traits) [21]. Out of the 10 items, 4 are used as “filler” items (i.e., not calculated in total score), while the other 6 are scored for optimism. Items on the LOT-R are rated on a 5-point Likert-type scale ranging from 1 (*I disagree a lot*) to 5 (*I agree a lot*) with higher scores indicating greater optimism (range 0–40). The alpha for this measure was .81.

#### Perceived coping abilities

The Measure of Current Status–A (MOCS-A) [22] is a 13-item measure that assesses perceived ability in utilizing coping skills (e.g., restructuring thoughts, relaxation, working through tension and stress). Items on the MOCS-A are scored on a 5-point Likert-type scale ranging from 0 (*I cannot do this at all)* to 4 (*I can do this extremely well*) with higher total scores representing greater perceived coping abilities (range 13–52). The alpha for this measure was .90.

#### Mindfulness

The Cognitive and Affective Mindfulness Scale (CAMS) [23] is a 12-item measure used to assess an individual’s mindfulness in the present-moment (i.e., nonavoidant and nonjudgmental thoughts and feelings). Items on the CAMS are scored on a 5-point Likert-type scale ranging from 0 *(not at all)* to 4 (*almost always*). Items are then summed with higher scores indicating greater mindfulness (range: 12–48). The alpha for this measure was .81.

#### Empathy

The Interpersonal Reactivity Index (IRI) [24] is a 28-item measure used to assess an individual’s dispositional empathy across four subscales: perspective taking, fantasy, empathic concern, and personal distress. Our study used one subscale from this measure, empathic concern, which is 7 items presented as a total score. Items on the IRI are scored on a 5-point Likert scale ranging from 0 *(does not describe me well)* to 4 *(describes me very well*; range: 0–28). The alpha for this measure was .81.

#### User experience

In addition to our resiliency outcomes, we assessed general user experience using non-validated questions delivered after program completion [25]. These questions pertained to program satisfaction and perceived financial value, had users not participated for free. The questions we asked included: “Generally speaking, I enjoyed participating in the NF-Web program”, “I found the NF-Web program to be helpful”, “I would recommend NF-Web to other people with NF”, and “If I had not participated for free as part of this research study, I would consider paying money to participate in this program (NF-Web)”. These questions were rated on a 5-point Likert-type scale from 0 (*Strongly Disagree)* to 4 (*Strongly Agree*) with 2 representing neutral responding (*Neither Agree or Disagree)*. Each individual item was scored and examined separately, with higher scores indicating greater subjective user experience satisfaction or perceived financial value. We examine these data based on frequency scores (*N*, %) instead of central tendencies (*M*, *SD*) for ease of interpretation.

#### Semi-structured exit interview

Within the month of program completion (0–4 weeks), participants scheduled and completed an exit interview over the telephone with the PI. Participants were asked questions related to their participation experience, including questions like, “What did you see as improving most from the program?” and “What did you think about the session materials?” Interviews were audio recorded and transcribed.

### Data analysis

We used the Statistical Packages for the Social Sciences version 25 software (SPSS 25) to analyze quantitative data similarly to the main 3RP-NF trial [11,14]. Specifically, we conducted a secondary data analysis to examine the changes in resiliency outcomes and user experience associated with NF-Web participation and used simple paired sample *t*-tests from pre- to post-test. In addition to these inferential tests, we also explored descriptive statistics for user experience surveys (*M*, *SD)*. These data are available in a Supporting Information file (below) as part of this manuscript.

For our qualitative data analysis, our study team examined and discussed each transcribed interview for rapid data analysis (RDA) [26] summaries similar to methods we have used in other qualitative studies [27]. Compared to traditional qualitative methods, rapid data analysis seeks to understand key insights or intervention features rather than provide a detailed, theoretically rich understanding of a concept. This approach allows study teams to acquire qualitative data on a shorter timeline than traditional qualitative methods that is readily applicable to real-world challenges [28–31]. Through our rapid data analysis, we were able to review the insights gained from each cohort and recognize when we stopped generating new conclusions from transcriptions. Notes were taken from each transcript as they related to three major categories: *General feedback*, *Specific positive aspects of the program*, and *Areas for program improvement*. Given that our qualitative findings were an exploratory outcome for our current study, the RDA procedure allowed us to quickly extract key aspects of each interview related to our specific areas of focus without the time and resource burden of a full thematic analysis used in previous NF studies [12].

## Results

### Sample characteristics and attrition

**Fig 1:**
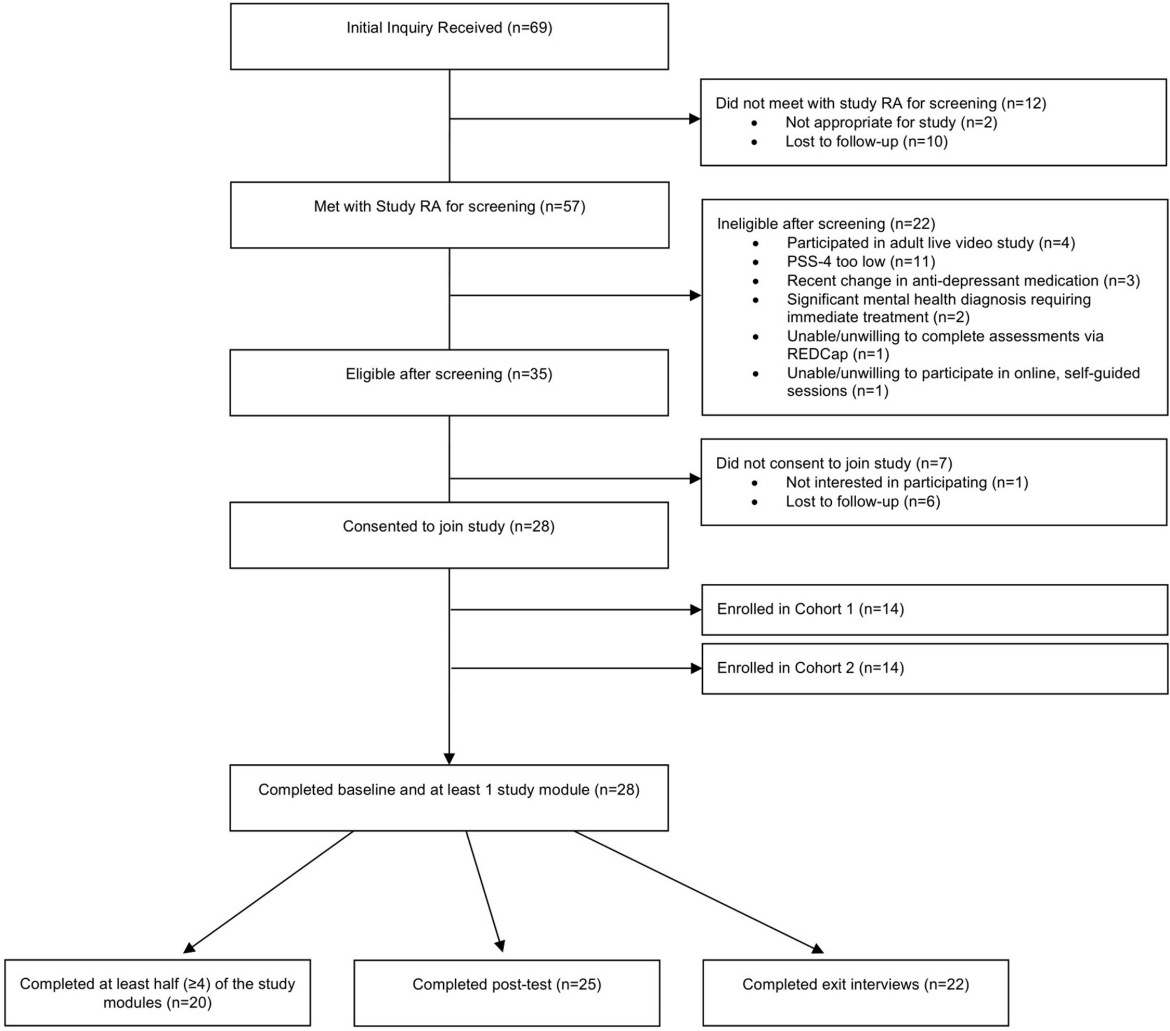
CONSORT diagram for NF-Web.

The sample demographics and attrition have been described in full in the primary outcomes paper [13]. Briefly, of the 57 adults screened for eligibility, 28 (14 per cohort; *N* = 28; 78.6% female, 82.1% white) met study criteria and enrolled. Of these, all 28 participants completed baseline assessments and at least one module of NF-Web (100%) and 20 participants completed at least half (≥4) of the modules (71%; treatment adherence). Of those enrolled, 25 participants completed post-test (89%). Screening and completion data are presented in Fig 1. Descriptively, there did not appear to be major differences between completers and non-completers. The sample included 19 participants with NF1 (67.86%), 6 with NF2 (21.43%), and 3 with SCHW (10.71%). Participant demographic variables are presented in Table 2, with unadjusted means for the resiliency outcomes at each time point presented in Table 3.

**Table 2:** Demographic characteristics for NF-Web sample (N = 28).

Characteristics		N (%)
Diagnosis		
NF1		19 (67.86%)
NF2		6 (21.43%)
Schwannomatosis		3 (10.71%)
Age		*M* = 45.86 (*SD* = 13.67)
Gender		
Female		22 (78.6%)
Male		6 (21.5%)
Learning diagnosis		
Yes, I Was Diagnosed with One		8 (28.57%)
I Think So, I Was Never Formally Diagnosed		4 (14.29%)
No		14 (50.00%)
I Don’t Know		2 (7.14%)
Education Level		
Completed high school		5 (17.9%)
Some college or associate degree		12 (42.9%)
Completed 4 years of college		6 (21.4%)
Graduated of Professional Degree		5 (17.9%)
Marital Status		
Married		14 (50.00%)
Living With Someone in a Committed Relationship		1 (3.57%)
Single		10 (35.71%)
Separated		1 (3.57%)
Divorced		1 (3.57%)
Widowed		1 (3.57%)
Race		
White		23 (82.14%)
Black/African American		1 (3.57%)
More Than One Race		2 (7.14%)
Missing/ choose not to answer		2 (7.14%)
Ethnicity		
Hispanic or Latino/Latina		2 (7.14%)
Not Hispanic or Latino/Latina		25 (89.29%)
Missing/ choose not to answer		1 (3.57%)

**Table 3:** Unadjusted baseline and post-test resiliency outcomes (M/SD) after NF-Web participation.

Measurement	Baseline (T1)	Post-test (T2)	*M Diff (T2-T1)*
**Social support**	71.39 (17.62)	76.48 (19.19)	5.09
**Gratitude**	32.29 (8.60)	35.04 (7.48)	2.75
**Optimism**	10.40 (3.48)	11.64 (4.46)	1.24
**Mindfulness**	29.29 (5.99)	33.54 (6.77)	4.25
**Empathy**	23.87 (4.01)	23.43 (4.46)	-0.43
**Perceived coping abilities**	23.00 (8.53)	29.27 (12.06)	6.27

### Post-intervention outcomes

Participation in NF-Web was associated with improvements in gratitude (*M*_*difference*_ = +2.75; 95% CI: -4.94 –-0.56; *p* = .016), coping (*M*_*difference*_ = +6.27; 95% CI: -10.72 –-1.82; *p* = .008), and mindfulness (*M*_*difference*_ = +4.25; 95% CI: -6.61 –-1.89; *p* = .001) from baseline to post-test. NF-Web was not associated with improvements in social support (*M*_*difference*_ = +5.09; 95% CI: -10.42–0.25; *p* = .061), optimism (*M*_*difference*_ = +1.24; 95% CI: -2.57–0.09; *p* = .067), or empathy (*M*_*difference*_ = -0.43; 95% CI: -1.19–2.06; *p* = .584).

### Exploratory findings

#### User experience data

User experience outcomes indicated that most participants enjoyed participating in NF-Web (“Agree” or “Strongly Agree”; *n* = 18; 72%), found the program helpful (“Agree” or “Strongly Agree”; *n* = 17; 68%), and would recommend NF-Web to a friend (“Agree” or “Strongly Agree”; *n* = 14; 56%). In terms of financial value (i.e., whether participants would have been willing to pay for NF-Web), a majority of participants were either ambivalent (“Neither agree nor disagree”; *n* = 11; 44%) or unwilling to pay for NF-Web (“Disagree” or “Strongly disagree”; *n* = 10; 40%) had it not been offered for free as part of this study. Of the three participants who would be willing to pay for NF-Web, none offered an amount they would be willing to pay.

#### Qualitative exit interview

Based on rapid data analyses, the general feedback we received reflected a predominant enjoyment of the program. Participants found the website easy to navigate and described the modules as useful. The self-guided, asynchronous nature of the program was convenient for participants who were able to complete session materials when their schedules allowed. Specific positive aspects of the program included user enjoyment of the video format, citing their preferences for audio-visual learning. Some participants found the lesson transcripts useful if they had hearing differences or if English was their second language. Many participants appreciated the gratitude skills session, with one participant reflecting, “you have more than you realize.” All participants enjoyed working with the study staff. Regarding areas of improvement, some study participants provided suggestions for NF-Web. The discussion board embedded into the website was not heavily used, and participants expressed interest in receiving reminders to engage with the forum to benefit from the group experience. Some users experienced variable video and sound quality or issues with the webpage on different devices (i.e., iPad). Some participants with mild NF symptoms described their experiences watching modules that did not apply to them, with some appreciating the perspective of the future and others requesting disclaimers that certain experiences are not universal. Last, many participants suggested adding patients with NF to the video modules for future versions of NF-Web.

## Discussion

We designed NF-Web for patients with NF who experienced barriers (i.e., transportation, mobility, schedule conflicts, learning differences, NF-specific physical appearance concerns) to traditional and virtual forms of care (live video groups). NF-Web was based on the live video adult 3RP-NF [11,14] and was adapted with participant feedback [12]. Our early feasibility trial [13] demonstrated that NF-Web is feasible, acceptable, and associated with improvements in QoL and emotional distress. Findings from the current study suggest that participation in NF-Web was associated with additional within-group improvements in gratitude, coping, and mindfulness from baseline to post-test. No significant differences were observed in social support, optimism, or empathy after program completion. Additionally, participants endorsed positive user experience and provided useful feedback during exit interviews for improving NF-Web.

Our findings were consistent with the live video 3RP-NF program, where participation in 3RP-NF was associated with improvements in coping and mindfulness compared to a health education placebo control group [14]. Coping is a multifaceted skill that has been demonstrated to reduce the cognitive impact of a difficult person-environment relationship [32] and improve health outcomes in other populations [33–35]. Additionally, mindfulness has been shown to reduce cognitive vulnerabilities through the increase of skillful response and awareness [36]. These resiliency outcomes are important targets for psychosocial interventions, as the ability to cope with diagnosis-related stressors and non-judgmentally manage symptoms and stress have been found to improve health outcomes in other populations [14,33–35]. We anticipated improvements in coping and mindfulness specifically because NF-Web directly targets and teaches these resiliency skills through engaging video lessons and homework assignments. These skills are amenable to psychosocial training [37] and have been shown to improve after participation in the 3RP program in other medical populations [11,14,37].

Different from the live video 3RP-NF, participation in NF-Web was associated with improvements in gratitude. Gratitude has been associated with significant improvements in positive affect and well-being [38,39], decreases in physical symptoms [40], and is a proximal treatment target of the 3RP-NF program [6]. Although this finding was encouraging as a signal of improvement, we are not able to draw definitive conclusions about this variable due to the lack of a comparison for this small feasibility trial. Future fully powered studies will assess changes in gratitude post-intervention and its potential for improvement after deliberate skills training.

Nonsignificant improvements in social support and empathy may be due to the asynchronous design of our program. NF-Web was developed to be a convenient, asynchronous web-based tool for people with NF. This unique characteristic of NF-Web may have impacted these interpersonally focused outcomes (i.e., social support and empathy), which are typically aided and stimulated by group interaction [41]. Interventions which offer in-person or live video interactions, like our 3RP-NF program [14], may be more likely to facilitate improvements in these interpersonally focused resiliency outcomes after participation. Future work may look to explore how interpersonal aspects of these programs can be better supported as part of self-guided programs like NF-Web (e.g., collaborative homework assignments, encouraging social media engagement).

It is possible that NF-Web did not result in significant improvements in optimism due to both the diversity of illness presentation (i.e., heterogeneity of symptoms and experiences) impacting one’s outlook on the future, as well as optimism being more related to dispositional factors which may be more trait-like vs. state-like. Research suggests that one’s optimism is more related to personal history of successes and internal attributes [19], rather that one’s environment and external attributes. Also, programs like NF-Web are relatively brief and may not have the “treatment potency” (i.e., strength) of intervention effect to directly improve an individual’s sense of optimism. The live video 3RP-NF also did not show improvements in optimism [14], and other interventions which target optimism have generally shown mixed results [42,43].

Based on our exploratory findings, NF-Web was well-received and deemed both helpful and enjoyable by users, and participants indicated that they would recommend the program to others with NF. Specifically for rare illness populations like NF, gathering information on user experience can be highly informative of the needs of the population [12]. Future interventions should reflect an incorporation of suggestions given by participants, to both optimize intervention results and promote user engagement. Given the diversity of illness experiences, we cannot assume that all participants will respond to the intervention in the same way, nor can we create an intervention that addresses all needs. Incorporating coaching on how to apply general skills to meet specific needs may benefit participants in future iterations of our program. Implicit measures of attention and engagement (e.g., screentime, clicks) may also be useful to collect in future interventions, as we may capture an added dimension of user attitudes through such measures. Additionally, we found that a majority of participants were either ambivalent or unwilling to pay for NF-Web, had it not been offered for free. Given that user perceptions of value and quality are often influenced by price [44], it is possible that offering free interventions may make participants less likely to consider paying for them in the future. Designing future iterations with payment requirements in mind may be beneficial to assist with financial support of these programs. Since this was an early feasibility study, we did not integrate product design aspects (e.g., marketing, aesthetics, advertising, pay walls, and free trial periods)–though they may be useful to optimize user experience and aid with producing revenue from this program.

The NF-Web study has several limitations and strengths. Study limitations included a modest sample size, lack of comparison group, a homogenous sample (white, well-educated, female), and limited follow-up assessment (one post-test timepoint). Additionally, we were not able to fully gauge user engagement (i.e., screentime tracking, video view counts, etc.) as the technology of NF-Web is still in initial phases of development. The accessibility and scalability of NF-Web for an underserved population, the opportunity for multiple modalities of intervention and assessment for people with NF, and the generally positive reception of the program and retention of participants are clear strengths and warrant more attention in future work related to NF-Web.

## Conclusion

Our study demonstrates that participants in NF-Web enjoyed the platform and its content. Despite the small sample size, we observed improvement in gratitude, coping, and mindfulness which are powerful resiliency outcomes that influence overall well-being, physical symptoms, and the reduction of cognitive vulnerabilities. Results support NF-web as a possible future option for people with NF who have barriers (i.e., transportation, mobility, schedule conflicts, learning differences, NF-specific physical appearance concerns) to participation in traditional support programs. Future directions should include exploring multifaceted learning approaches to interventions (i.e., audio, audio-visual), as well as incorporating interpersonal engagement opportunities in asynchronous formats to promote participant retention. With participants able to revisit modules whenever is convenient, NF-web’s asynchronous format provides an essential foundation for exploring many temporal and comprehensible advantages to a diversity of learners in future interventions.

## Supporting information

S1 Fig About NF-Web webpage. (TIFF)

S2 Fig NF-Web discussion board. (TIFF)

S3 Fig NF-Web module. (TIFF)

S4 Fig NF-Web homepage. (TIFF)

S1 File Quantitative dataset. (ZIP)

## References

[1] Tamura R. Current Understanding of Neurofibromatosis Type 1, 2, and Schwannomatosis. Int J Mol Sci. 2021;22(11). Epub 20210529. doi: 10.3390/ijms22115850 ; PubMed Central PMCID: PMC8198724. 34072574 PMC8198724

[2] Evans DG, Howard E, Giblin C, Clancy T, Spencer H, Huson SM, et al. Birth incidence and prevalence of tumor-prone syndromes: estimates from a UK family genetic register service. Am J Med Genet A. 2010;152a(2):327–32. doi: 10.1002/ajmg.a.33139 . 20082463

[3] Lu-Emerson C, Plotkin SR. The neurofibromatoses. Part 2: NF2 and schwannomatosis. Rev Neurol Dis. 2009;6(3):E81–6. . 19898272

[4] Farschtschi S, Mautner VF, McLean ACL, Schulz A, Friedrich RE, Rosahl SK. The Neurofibromatoses. Dtsch Arztebl Int. 2020;117(20):354–60. doi: 10.3238/arztebl.2020.0354 ; PubMed Central PMCID: PMC7373809. 32657748 PMC7373809

[5] de Blank PMK, Gross AM, Akshintala S, Blakeley JO, Bollag G, Cannon A, et al. MEK inhibitors for neurofibromatosis type 1 manifestations: Clinical evidence and consensus. Neuro-Oncology. 2022;24(11):1845–56. doi: 10.1093/neuonc/noac165 35788692 PMC9629420

[6] Vranceanu AM, Merker VL, Park E, Plotkin SR. Quality of life among adult patients with neurofibromatosis 1, neurofibromatosis 2 and schwannomatosis: a systematic review of the literature. J Neurooncol. 2013;114(3):257–62. Epub 20130702. doi: 10.1007/s11060-013-1195-2 . 23817811

[7] Wang DL, Smith KB, Esparza S, Leigh FA, Muzikansky A, Park ER, et al. Emotional functioning of patients with neurofibromatosis tumor suppressor syndrome. Genet Med. 2012;14(12):977–82. Epub 20120809. doi: 10.1038/gim.2012.85 ; PubMed Central PMCID: PMC3982605. 22878510 PMC3982605

[8] Wolkenstein P, Zeller J, Revuz J, Ecosse E, Leplège A. Quality-of-life impairment in neurofibromatosis type 1: a cross-sectional study of 128 cases. Arch Dermatol. 2001;137(11):1421–5. doi: 10.1001/archderm.137.11.1421 . 11708944

[9] Page PZ, Page GP, Ecosse E, Korf BR, Leplege A, Wolkenstein P. Impact of neurofibromatosis 1 on Quality of Life: a cross-sectional study of 176 American cases. Am J Med Genet A. 2006;140(18):1893–8. doi: 10.1002/ajmg.a.31422 . 16906549

[10] Kodra Y, Giustini S, Divona L, Porciello R, Calvieri S, Wolkenstein P, et al. Health-related quality of life in patients with neurofibromatosis type 1. A survey of 129 Italian patients. Dermatology. 2009;218(3):215–20. Epub 20081217. doi: 10.1159/000187594 . 19088462

[11] Vranceanu AM, Riklin E, Merker VL, Macklin EA, Park ER, Plotkin SR. Mind-body therapy via videoconferencing in patients with neurofibromatosis: An RCT. Neurology. 2016;87(8):806–14. Epub 20160722. doi: 10.1212/WNL.0000000000003005 . 27449066

[12] Lester EG, Popok PJ, Grunberg VA, Baez A, Herrawi F, Vranceanu AM. Stopping to Listen: Using Qualitative Methods to Inform a Web-Based Platform for Adults With Neurofibromatosis. J Patient Exp. 2021;8:23743735211049644. Epub 20211126. doi: 10.1177/23743735211049644 ; PubMed Central PMCID: PMC8640297. 34869834 PMC8640297

[13] Lester EG, Fishbein NS, Peterson A, Vranceanu A-M. Early feasibility testing of a web-based mind-body resiliency program for adults with neurofibromatosis: The NF-Web study. PEC Innovation. 2022;1:100076. doi: 10.1016/j.pecinn.2022.100076 37213775 PMC10194129

[14] Zale EL, Pierre-Louis C, Macklin EA, Riklin E, Vranceanu AM. The impact of a mind-body program on multiple dimensions of resiliency among geographically diverse patients with neurofibromatosis. J Neurooncol. 2018;137(2):321–9. Epub 20171223. doi: 10.1007/s11060-017-2720-5 . 29275505

[15] Harris PA, Taylor R, Thielke R, Payne J, Gonzalez N, Conde JG. Research electronic data capture (REDCap)—a metadata-driven methodology and workflow process for providing translational research informatics support. J Biomed Inform. 2009;42(2):377–81. Epub 20080930. doi: 10.1016/j.jbi.2008.08.010 ; PubMed Central PMCID: PMC2700030. 18929686 PMC2700030

[16] Lester EG, Hopkins SW, Popok PJ, Vranceanu AM. Adaptation of a Live Video Mind-Body Program to a Web-Based Platform for English-Speaking Adults With Neurofibromatosis: Protocol for the NF-Web Study. JMIR Res Protoc. 2021;10(6):e27526. Epub 20210610. doi: 10.2196/27526 ; PubMed Central PMCID: PMC8262670. 34110294 PMC8262670

[17] Sherbourne CD, Stewart AL. The MOS social support survey. Soc Sci Med. 1991;32(6):705–14. doi: 10.1016/0277-9536(91)90150-b . 2035047

[18] McCullough ME, Emmons RA, Tsang JA. The grateful disposition: a conceptual and empirical topography. J Pers Soc Psychol. 2002;82(1):112–27. doi: 10.1037//0022-3514.82.1.112 . 11811629

[19] Scheier MF, Carver CS. Optimism, coping, and health: assessment and implications of generalized outcome expectancies. Health Psychol. 1985;4(3):219–47. doi: 10.1037//0278-6133.4.3.219 . 4029106

[20] Scheier MF, Carver CS, Bridges MW. Distinguishing optimism from neuroticism (and trait anxiety, self-mastery, and self-esteem): a reevaluation of the Life Orientation Test. J Pers Soc Psychol. 1994;67(6):1063–78. doi: 10.1037//0022-3514.67.6.1063 . 7815302

[21] Glaesmer H, Rief W, Martin A, Mewes R, Brähler E, Zenger M, et al. Psychometric properties and population-based norms of the Life Orientation Test Revised (LOT-R). Br J Health Psychol. 2012;17(2):432–45. Epub 20110721. doi: 10.1111/j.2044-8287.2011.02046.x . 22106985

[22] Carver C. Measure of Current Status Department of Psychology, University of Miami [Internet]. 2006 [cited 2022 June 20]. Available from: http://local.psy.miami.edu/faculty/ccarver/sclMOCS.phtml

[23] Feldman G, Hayes A, Kumar S, Greeson J, Laurenceau J-P. Mindfulness and Emotion Regulation: The Development and Initial Validation of the Cognitive and Affective Mindfulness Scale-Revised (CAMS-R). Journal of Psychopathology and Behavioral Assessment. 2007;29(3):177–90. doi: 10.1007/s10862-006-9035-8

[24] Davis M. A Multidimensional Approach to Individual Differences in Empathy. JSAS Catalog of Selected Documents in Psychology. 1980;10:85.

[25] Schrepp M, Hinderks A, Thomaschewski J. Construction of a Benchmark for the User Experience Questionnaire (UEQ). International Journal of Interactive Multimedia and Artificial Intelligence. 2017;4(4):40–4.

[26] Lewinski AA, Crowley MJ, Miller C, Bosworth HB, Jackson GL, Steinhauser K, et al. Applied Rapid Qualitative Analysis to Develop a Contextually Appropriate Intervention and Increase the Likelihood of Uptake. Med Care. 2021;59(Suppl 3):S242–s51. doi: 10.1097/MLR.0000000000001553 ; PubMed Central PMCID: PMC8132894. 33976073 PMC8132894

[27] Reichman M, Bakhshaie J, Grunberg VA, Doorley JD, Vranceanu AM. What Are Orthopaedic Healthcare Professionals’ Attitudes Toward Addressing Patient Psychosocial Factors? A Mixed-Methods Investigation. Clin Orthop Relat Res. 2022;480(2):248–62. doi: 10.1097/CORR.0000000000002043 ; PubMed Central PMCID: PMC8747600. 34779793 PMC8747600

[28] Gale RC, Wu J, Erhardt T, Bounthavong M, Reardon CM, Damschroder LJ, et al. Comparison of rapid vs in-depth qualitative analytic methods from a process evaluation of academic detailing in the Veterans Health Administration. Implementation Science. 2019;14(1):11. doi: 10.1186/s13012-019-0853-y 30709368 PMC6359833

[29] Taylor B, Henshall C, Kenyon S, Litchfield I, Greenfield S. Can rapid approaches to qualitative analysis deliver timely, valid findings to clinical leaders? A mixed methods study comparing rapid and thematic analysis. BMJ Open. 2018;8(10):e019993. Epub 20181008. doi: 10.1136/bmjopen-2017-019993 ; PubMed Central PMCID: PMC6194404. 30297341 PMC6194404

[30] Watkins DC. Rapid and Rigorous Qualitative Data Analysis:The “RADaR” Technique for Applied Research. International Journal of Qualitative Methods. 2017;16(1):1609406917712131. doi: 10.1177/1609406917712131

[31] Brown-Johnson C, Safaeinili N, Zionts D, Holdsworth LM, Shaw JG, Asch SM, et al. The Stanford Lightning Report Method: A comparison of rapid qualitative synthesis results across four implementation evaluations. Learn Health Syst. 2020;4(2):e10210. Epub 20191221. doi: 10.1002/lrh2.10210 ; PubMed Central PMCID: PMC7156867. 32313836 PMC7156867

[32] Lazarus R, Folkman S. Stress, Appraisal, and Coping: Springer; 1984.

[33] Grossman P, Niemann L, Schmidt S, Walach H. Mindfulness-based stress reduction and health benefits. A meta-analysis. J Psychosom Res. 2004;57(1):35–43. doi: 10.1016/S0022-3999(03)00573-7 . 15256293

[34] Penley JA, Tomaka J, Wiebe JS. The association of coping to physical and psychological health outcomes: a meta-analytic review. J Behav Med. 2002;25(6):551–603. doi: 10.1023/a:1020641400589:1020641400589 12462958

[35] Wang HH, Wu SZ, Liu YY. Association between social support and health outcomes: a meta-analysis. Kaohsiung J Med Sci. 2003;19(7):345–51. doi: 10.1016/S1607-551X(09)70436-X . 12926520 PMC11918198

[36] Bishop SR, Lau M, Shapiro S, Carlson L, Anderson ND, Carmody J, et al. Mindfulness: A Proposed Operational Definition. Clinical Psychology: Science and Practice. 2004;11(3):230–41. doi: 10.1093/clipsy.bph077

[37] Vranceanu AM, Merker VL, Plotkin SR, Park ER. The relaxation response resiliency program (3RP) in patients with neurofibromatosis 1, neurofibromatosis 2, and schwannomatosis: results from a pilot study. J Neurooncol. 2014;120(1):103–9. Epub 20140715. doi: 10.1007/s11060-014-1522-2 . 25022450

[38] Seligman ME, Steen TA, Park N, Peterson C. Positive psychology progress: empirical validation of interventions. Am Psychol. 2005;60(5):410–21. doi: 10.1037/0003-066X.60.5.410 . 16045394

[39] Wood AM, Froh JJ, Geraghty AW. Gratitude and well-being: a review and theoretical integration. Clin Psychol Rev. 2010;30(7):890–905. Epub 20100320. doi: 10.1016/j.cpr.2010.03.005 . 20451313

[40] Hill PL, Allemand M, Roberts BW. Examining the Pathways between Gratitude and Self-Rated Physical Health across Adulthood. Pers Individ Dif. 2013;54(1):92–6. doi: 10.1016/j.paid.2012.08.011 ; PubMed Central PMCID: PMC3489271. 23139438 PMC3489271

[41] Chen Y, Xu Y. Exploring the Effect of Social Support and Empathy on User Engagement in Online Mental Health Communities. Int J Environ Res Public Health. 2021;18(13). Epub 20210626. doi: 10.3390/ijerph18136855 ; PubMed Central PMCID: PMC8296998. 34206719 PMC8296998

[42] Kleiman EM, Chiara AM, Liu RT, Jager-Hyman SG, Choi JY, Alloy LB. Optimism and well-being: a prospective multi-method and multi-dimensional examination of optimism as a resilience factor following the occurrence of stressful life events. Cogn Emot. 2017;31(2):269–83. Epub 20151111. doi: 10.1080/02699931.2015.1108284 ; PubMed Central PMCID: PMC5215689. 26558316 PMC5215689

[43] Geers AL, Wellman JA, Helfer SG, Fowler SL, France CR. Dispositional optimism and thoughts of well-being determine sensitivity to an experimental pain task. Ann Behav Med. 2008;36(3):304–13. Epub 20081209. doi: 10.1007/s12160-008-9073-4 . 19067097

[44] Zeithaml VA. Consumer Perceptions of Price, Quality, and Value: A Means-End Model and Synthesis of Evidence. Journal of Marketing. 1988;52(3):2–22. doi: 10.2307/1251446

