# Supplementary figures and images for: Resiliency outcomes after participation in an asynchronous web-based platform for adults with neurofibromatosis: The NF-Web study
Source: PLoS One. 2023 Dec 21;18(12):e0295546. doi: 10.1371/journal.pone.0295546 (PMC10735002; doi:10.1371/journal.pone.0295546)

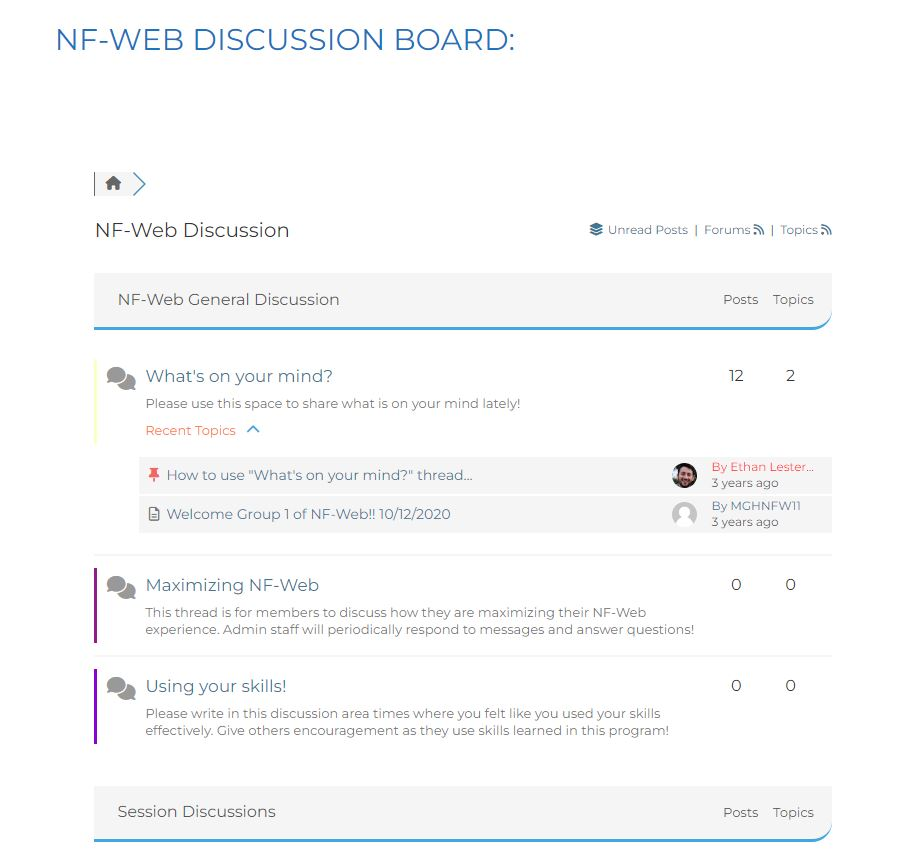

Supplement: S2 Fig — (TIFF) [file pone.0295546.s002.tiff]

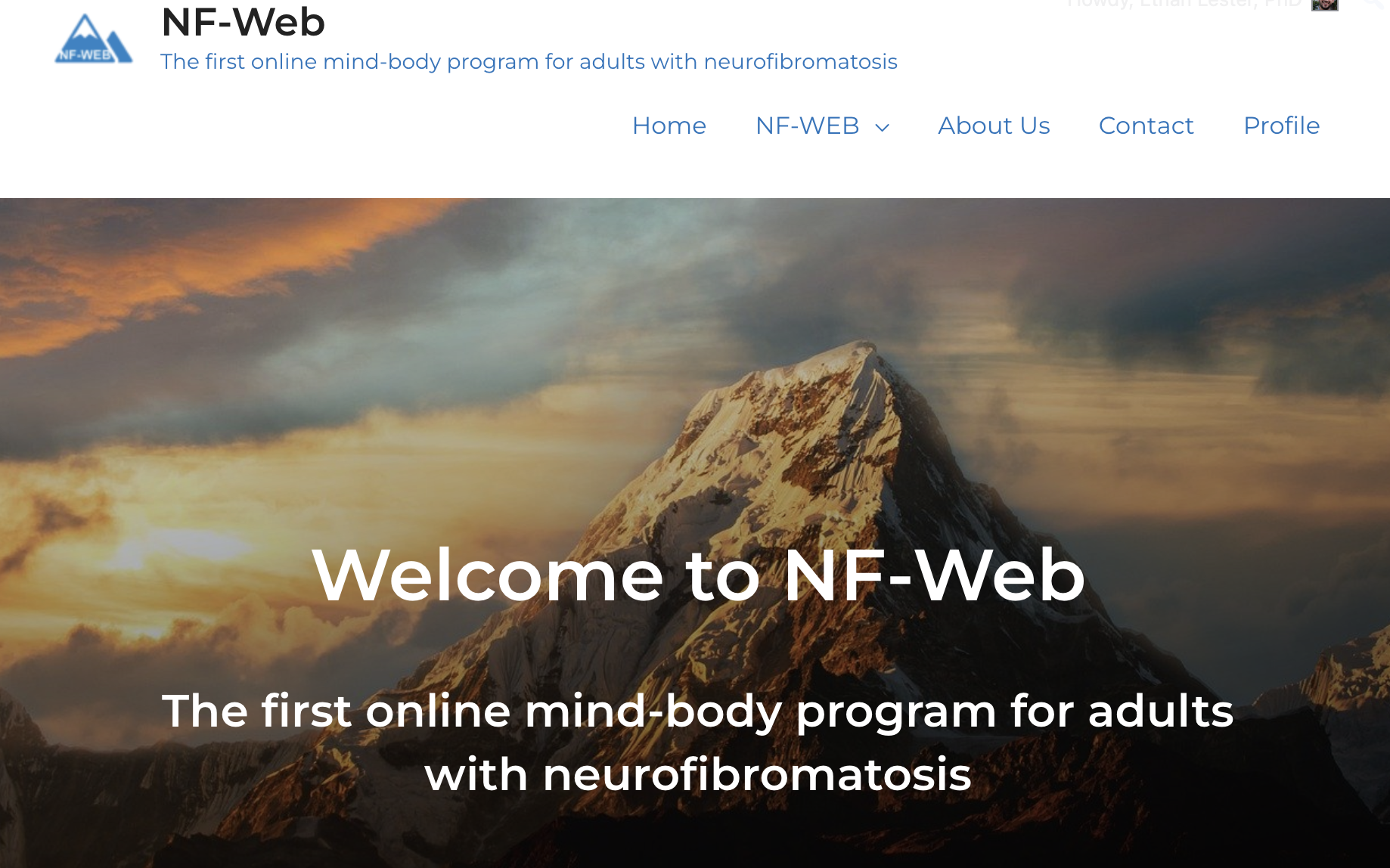

Supplement: S4 Fig — (TIFF) [file pone.0295546.s004.tiff]
